# A Psychophysical Imaging Method Evidencing Auditory Cue Extraction during Speech Perception: A Group Analysis of Auditory Classification Images

**DOI:** 10.1371/journal.pone.0118009

**Published:** 2015-03-17

**Authors:** Léo Varnet, Kenneth Knoblauch, Willy Serniclaes, Fanny Meunier, Michel Hoen

**Affiliations:** 1 Lyon Neuroscience Research Center, CNRS UMR 5292, Auditory Language Processing (ALP) research group, Lyon, France; 2 Stem Cell and Brain Research Institute, INSERM U 846, Integrative Neuroscience Department, Bron, France; 3 Laboratoire sur le Langage le Cerveau et la Cognition, CNRS UMR 5304, Auditory Language Processing (ALP) research group, Lyon, France; 4 INSERM U1028, Lyon Neuroscience Research Center, Brain Dynamics and Cognition Team, Lyon, France; 5 Université de Lyon, Université Lyon 1, Lyon, France; 6 Université Libre de Bruxelles, UNESCOG, CP191, Bruxelles, Belgique; Kyoto University, JAPAN

## Abstract

Although there is a large consensus regarding the involvement of specific acoustic cues in speech perception, the precise mechanisms underlying the transformation from continuous acoustical properties into discrete perceptual units remains undetermined. This gap in knowledge is partially due to the lack of a turnkey solution for isolating critical speech cues from natural stimuli. In this paper, we describe a psychoacoustic imaging method known as the Auditory Classification Image technique that allows experimenters to estimate the relative importance of time-frequency regions in categorizing natural speech utterances in noise. Importantly, this technique enables the testing of hypotheses on the listening strategies of participants at the group level. We exemplify this approach by identifying the acoustic cues involved in da/ga categorization with two phonetic contexts, Al- or Ar-. The application of Auditory Classification Images to our group of 16 participants revealed significant critical regions on the second and third formant onsets, as predicted by the literature, as well as an unexpected temporal cue on the first formant. Finally, through a cluster-based nonparametric test, we demonstrate that this method is sufficiently sensitive to detect fine modifications of the classification strategies between different utterances of the same phoneme.

## Introduction

In speech perception, we unconsciously process a continuous auditory stream with a complex time-frequency structure that does not contain fixed, highly reproducible, or evident boundaries between the different perceptual elements that we detect in the stream of speech. Phonemes [1] or syllables [2], the building-blocks of speech, are sophisticated perceptual entities. Through a long evolutionary process, human brains have learned to extract certain auditory primitives from the speech signal and associate them with different perceptual categories. For example, we perceive the sounds /d/ or /g/ as discrete and distinct elements, without being aware of the underlying perceptual construction causing their distinction [3,4]. Which acoustic features are extracted and used to perceive speech remains unknown, largely because of the lack of an experimental method enabling the direct visualization of auditory cue extraction. The aim of this paper is to propose and demonstrate the validity of adapting the classification image framework to directly visualize auditory functional cues actually used by individual listeners that are processing speech.

### Acoustic cues for speech perception

Speech is a continuous waveform comprising an alternation of harmonic and non-harmonic acoustic segments. Periodic sounds are caused by vibrations of the vocal folds and are shaped by resonances of the vocal tract to produce formants in the acoustic signal [5]. Thus, formants correspond to local energy maxima inside the spectral envelope of the signal and are present for vocalic sounds (*e*.*g*., vowels such as /a/ or /u/) or voiced consonants (*e*.*g*., /v/, /d/, or /g/). The number of formants is typically 4 to 5, depending on the phoneme considered. Formants cover a frequency range from approximately 150 Hz to 4 to 6 kHz, with approximately one formant per kHz, and last approximately 100 ms. Each vowel appears to be loosely tied to a specific formantic structure (essentially determined by the height of the first two formants, F1 and F2). Perturbations of the acoustic flux created by the rapid occlusion or release of the air flow generate silences, hisses, bursts or explosions that constitute the core of consonantal sounds (*e*.*g*., consonants such as /t/, /p/ or /sh/). Their presence transitorily inflects the formant trajectories, thus creating brief formantic transitions. The formantic structure and formant dynamics are examples of spectrotemporal acoustic cues that could be exploited at the acoustic/phonetic interface [6]. By studying coarticulated utterances of /alda/, /alga/, /arda/, and /arga/, [7] determined that (1) the identity of the first consonant affected the spectral content of the second syllable, and vice-versa, and that (2) listeners were able to compensate for this coarticulation during perception. Although the first effect is clearly due to the partial overlapping of articulatory commands between adjacent phonemes, the exact nature of the compensation phenomenon remains undetermined [8–11]. Coarticulation introduces internal variations into the system referred to as allophonic variations: a range of different formantic structures will be perceived as the same phoneme. This phenomenon makes the system more resistant to intra- and inter-speaker variations, but it also makes the problem of learning to associate acoustic cues to phonemic percepts more difficult and the reverse engineering problem of designing automatic speech recognition and automatic speech comprehension systems largely unresolved [12].

### Identifying auditory primitives for speech perception

The precise mechanism underlying the transformation from continuous acoustical properties into the presence or absence of some acoustic cues and finally into a discrete perceptual unit remains undetermined. The acoustic-phonetic interface has been studied extensively since 1950. Many studies on this topic have been conducted under experimental conditions, which have involved stimuli that were degraded in a controlled fashion in order to narrow the problem to a small number of possible cues. Among the most well-known attempts is the series of papers published by the Haskins Laboratories on the relationship between second formant transition and stop consonant perception using synthetic speech [13,14]. However, their conclusions are inherently limited by the non-naturalness of the synthesized stimuli: the variations of synthetic stimuli are restricted to a small number of cues, and thus they may not be processed in the same manner as natural stimuli. Furthermore, participants exposed to this type of stimuli often report them as “unnatural” and typically achieve lower recognition performances, a clear sign that the natural cues are poorly represented in synthesized speech. More recent work has also relied on drastic spectral and/or temporal impoverishment of the speech signal [15,16]. However, in a “real-life” situation, listeners are not typically required to address filtered speech but have access to the entire spectrum. As before, the question remains: Are the evidenced acoustic cues with synthetic speech identical to those for natural speech? The resistance of speech intelligibility to drastic signal reductions, such as those noted above, could rely on secondary perceptual cues not used in natural listening situations. Scientists seeking to address this problem will ultimately be required to use natural speech production as stimuli.

In this context, a recent solution demonstrates the merits of using a masking noise on natural speech utterances to isolate the regions of the spectrogram crucial for identifying a particular phoneme. The technique initially proposed by [17] involves masking natural speech utterances with noise at various signal-to-noise ratios (SNRs). By analyzing the patterns of confusion in a participant’s responses with respect to the noise level, researchers were able to identify the point at which noise masks the crucial acoustic cue, thus corresponding to a drop of correct identifications [18,19].

Alternative approaches for determining the mapping of sounds to mental representations of acoustic cues have been enabled by recent statistical developments in neuroimaging, including advances in the multivariate encoding/decoding models of neural activity. By reverse-engineering the processing of speech in the brain, it has become possible to reveal the encoding of sub-phonological information in the auditory cortex [20,21]. One such solution has been to record the firing rate modulations of individual auditory neurons in response to specific stimuli to derive their spectrotemporal receptive-fields (STRFs), which are a linear approximation of the time-frequency function of the neuron. This technique has been widely used in studying birds, specifically when hearing conspecific birdsongs [22,23]. These studies have demonstrated that auditory neurons are tuned to specific time-frequency regions, surrounded by one or more inhibitory regions. Spectrotemporal filters are assumed to be somewhat similar for human auditory neurons. Electrocorticographical (ECoG) recordings have enabled the estimation of average STRFs for small groups of human auditory neurons in epileptic patients [24], thereby strengthening the idea that the basic auditory cues for humans are also composed of an excitatory region surrounded by inhibitory regions. As a next step, [20] gathered STRFs from clusters of neurons that are functionally similar, e.g., auditory neurons responding preferentially to particular phonemes. They obtained the first images of the encoding of acoustic cues for several features, as well as the tuning of neurons to frequencies corresponding to formant values. Although these results represent a major breakthrough in understanding how speech sounds are primarily decoded along the primary auditory pathway, it is difficult to infer how this information is combined to facilitate the identification of one phoneme rather than another phoneme. Computational models have been proposed [25] that link the STRF with a multiresolution representation of speech sounds in the auditory cortex. This approach could provide a unified model of the transformation of a speech input from the cochlea to the midbrain. However, this account currently remains theoretical, because of the lack of a method allowing the observation of the use of acoustic cues in normal participants and other non-epileptic patients and large-group studies or studies on the individual variations of these processes.

### The auditory classification image approach

In a previous paper [26], we demonstrated the feasibility of addressing this gap in the auditory domain by adapting a method designed to identify the primitives of simple perceptual tasks, the classification image technique. Inspired from an auditory tone-in-noise detection experiment by Ahumada and Lovell [27], classification images have then been developed in the visual domain and successfully used to study Vernier acuity [28], perceptual learning [29,30], the interpolation of illusory contours [31], the detection of luminance [32] and chromatic [33] modulations, and recently face pareidolia [34]. We developed the Auditory Classification Image (ACI) technique by transposing this experimental and statistical framework to an auditory categorization task between two target speech sounds (/aba/ and /ada/). The two signals were presented in an additive Gaussian noise, and participants were asked to indicate whether the target was /aba/ or /ada/. Each participant’s response was then linked to the specific noise configuration in the corresponding trial with a Generalized Linear Model (GLM) with smoothness priors. The rationale underlying this technique is that if the time-frequency coordinates at which the noise interferes with the decision of the observer are known, then the regions on which the observer focuses to perform the task would also be known. By fitting the decision weights corresponding to every pixel of the representation, it became possible to draw a time-frequency map of the categorization strategy and directly visualize which parts of the stimulus are crucial for the decision.

In the first report on ACIs, we only reported individual data on three volunteers and used two speech productions as targets, thus leaving the question of the specificity of the obtained ACIs to these particular utterances unanswered. In the present study, we aimed to 1) further develop the method and complete a first group study to extend the feasibility of the method to group studies; 2) apply statistical tests permitting the evaluation of statistical significance inside or between classification images and 3) explore the specificity of the ACI to the utterances used as targets. To this end, we acquired auditory classification images from a group of 16 participants performing 10,000 categorizations of the four /alga/, /alda/, /aʁga/, /aʁda/ speech sounds.

## Materials and Methods

### Participants

Seventeen native speakers of French with no background knowledge of phonetics and phonology participated in this study. All participants had normal hearing, as confirmed by a standard pure-tone audiometric test (<20 dB HL, 125–8,000 Hz), and reported no history of neurological or developmental disorders. Additionally, participants were administered a series of tests on nonverbal intelligence (Raven’s Standard Progressive Matrices) and phonological and reading skills (ECLA-16+). They obtained scores within normal ranges on all tests (S1 Table). The study was approved by the Comité d'évaluation éthique de l'Inserm / Institutional Review Board (IORG0003254, FWA00005831, IRB00003888). Participants provided written informed consent before participating in the experiment, and they received financial compensation (€100) for their participation. One participant’s data were excluded from further analyses due to extremely poor performances that suggested the participant had misunderstood the instructions. Thus, the analyses are based on the answers of 16 participants (12 females; mean age: 22.6 years ± 4.6 years S.D).

### Stimuli

Four speech samples, i.e., /alda/, /alga/, /aʁda/, and /aʁga/, were recorded from one male speaker in a soundproof chamber at a sample rate of 48 kHz. The 4 stimuli were obtained by removing the silent gap between the two syllables to align the onset of the second syllable at the same temporal position and then equating the 4 sounds in root mean square and in duration (680 ms). The resulting speech signals (hereafter denoted $\underset{\_}{t}$) sounded perfectly natural and were perfectly intelligible in a quiet setting.

**Fig 1:**
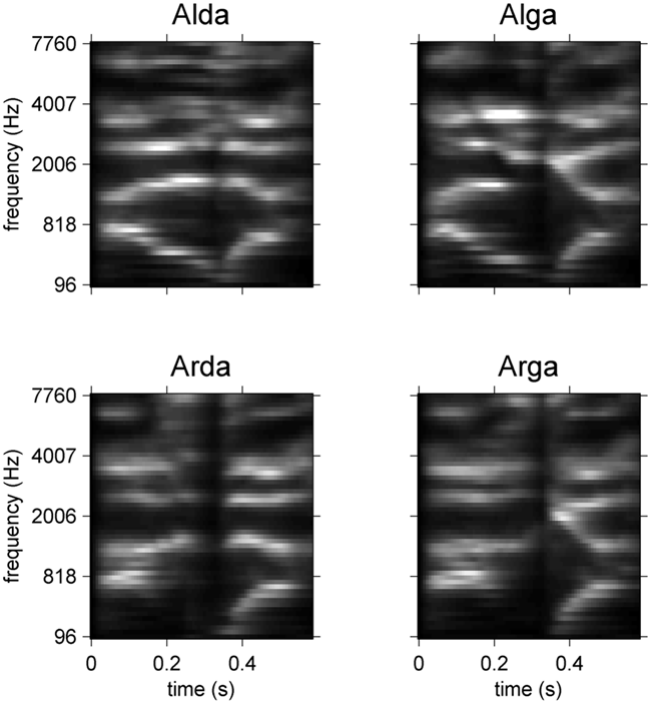
Cochleograms of the four stimuli involved in the experiment. Parameters for spectral and temporal resolution are identical to those used to derive the ACIs (see details in the main text).

Each stimulus $\underset{\_}{s}$ in this experiment consisted of one target signal $\underset{\_}{t}$ embedded in an additive Gaussian noise $\underset{\_}{n}$ at a given SNR using Equation (1).

$${\underset{\_}{s}}_{i}=\alpha_{i}\cdot {\underset{\_}{t}}_{k_{i}}+{\underset{\_}{n}}_{i}$$ (1)

where *i* is the trial number; *k_i_* the signal number associated with this trial; and *α_i_* a factor determining the SNR during the course of the experiment ($\alpha_{i}=10^{\frac{SNR_{i}}{20}}$, for ${\underset{\_}{n}}_{i}$ and ${\underset{\_}{t}}_{k_{i}}$ both normalized in power and SNR in dB). The sampling rate of the stimuli was set to 48 kHz for the original sounds. All stimuli were root-mean-square normalized and were then preceded by 75 ms of Gaussian-noise with a Gaussian fade-in to avoid abrupt attacks. The cochleograms of the 4 stimuli are shown in Fig. 1.

### Experimental procedure

Participants were seated in a sound booth in front of a computer monitor and wore Sennheiser’s HD 448 headphones. They completed a set of 10,000 trials consisting of 2,500 noisy presentations of each of the 4 speech signals, presented in random order. For each trial, they were asked to listen carefully and then to indicate, by a button press, whether the final syllable was ‘da’ or ‘ga’. The response to trial *i* is denoted *r_i_*(= 0 for ‘da’ and 1 for ‘ga’), and the correct answer (corresponding to the target actually presented) is denoted *c*. Participants were allowed to replay the stimulus before entering their response. For each trial, the participant’s response, his/her response time, the SNR level, and the time-frequency configuration of noise ${\underset{\_}{n}}_{i}$ were recorded for offline analysis.

Given the long duration of the experiment (approximately 4 h), we divided it into 20 sessions of 500 trials completed over 4 days to avoid mental and auditory fatigue. Sessions were separated by minimum breaks of 3 min. In addition, there was a short practice block before the beginning of the experiment that was similar to the test phase, except that the correct answers were displayed after each trial. Over the course of the experiment, the SNR was adapted from trial-to-trial based on the participant’s responses by a 3-down 1-up staircase procedure [35], thereby allowing us to constantly target the 79% correct point on the psychometric function. The SNR was increased by one step after each incorrect response and decreased by one step after three consecutive correct responses from the last change in stimulus intensity. At the beginning of each session, the step size was set to 2 dB to accelerate convergence and then decreased 10% by each step until a step size of 0.2 dB was attained. The initial SNR level was -11 dB, and each session began with the final SNR of the previous session.

## Generating Auditory Classification Images

The method used for deriving ACIs has been previously detailed [26]. A summary is provided below, with a focus on several improvements that have been introduced since the publication of the first version.

### Cochleograms

The same preprocessing was applied to all noise and speech sounds before analysis. Cochleograms were generated from the waveforms using Lyon’s Cochlea Model [36], implemented in the classic Matlab Auditory Toolbox by Malcom Slaney (https://engineering.purdue.edu/~malcolm/interval/1998-010/). This algorithm involves a bank of simulated auditory filters of constant quality factor (Q = 8), spaced quasi-logarithmically and overlapping by 40% (this step factor was chosen to be slightly greater than the default parameter proposed by Slaney to ensure sufficient spectral resolution). The vertical axis of the cochleogram represents the center frequencies of each auditory filter. Two additional processing levels are implemented in this function to mimic the non-linear behavior of the hair cells: a half-wave rectifier followed by an Automatic Gain Control modeling the neural adaptation mechanism and a difference between adjacent filters to improve the frequency response. Finally, the output of the model is decimated in time to a sample rate of 64.1 Hz, with a time step of 15.6 ms. The cochleograms of our 4 stimuli are presented in Fig. 1. The cochleogram of the noise sound at each trial *i* was calculated and will be hereafter denoted by ${\underset{\_}{X}}_{i}$ in its vectorized form.

### Generalized Linear Model

For each participant, several ACIs were derived from the responses to all or part of the 10,000 trials using a GLM. This statistical model links the probability that the participant responded with 1, *P*(*r_i_* = 1), with the specific configuration of the noise through the following equation:

$$P\left(r_{i}=1\right)=\Phi \left({\underset{\_}{X}}_{i}{}^{T}\cdot \underset{\_}{\beta }+b_{c_{i}}\right).$$ (2)

where Φ a psychometric function (here, the inverse of the logit function); $\underset{\_}{\beta }$ the decision template; and $\underset{\_}{b}$ a two-level factor reflecting the influence of the target actually presented on the response. Phoneme categorization is regarded in this context as a simple template-matching process between the input sound and two mental representations of the targets stored in memory. The decision template corresponds to a particular linear weighting of the noise profile and is specific to the two targets involved in the task. The output of the dot-product ${\underset{\_}{X}}_{i}{}^{T}\cdot \underset{\_}{\beta }$ is added to the factor $\underset{\_}{b}$ to yield a linear predictor that is eventually transformed nonlinearly through the psychometric function into a probability ranging between 0 and 1. It is important to note that the GLM does not simulate the internal processing of the human speech perception system. However, it is useful for determining which variations of the stimulus affect human perception. Thus, our main goal was to approach the decision template $\underset{\_}{\beta }$ with an estimator $\hat{\underset{\_}{\beta }}$, the ACI.

### Smoothness priors

Rather than directly estimating the model parameters $\underset{\_}{\theta }=\left\{\underset{\_}{\beta },\underset{\_}{b}\right\}$ with a simple maximization of the log-likelihood $L\left(\underset{\_}{\theta }\right)=\log\left(P\left(\underset{\_}{r}|\underset{\_}{\theta },\underset{\_}{c},\underset{\_}{\underset{\_}{X}}\right)\right)$, we introduced a smoothness prior during the optimization of the GLM. This statistical development, named “Penalized Likelihood,” or “Generalized Additive Model” (GAM), has been widely used for estimating receptive fields at the neuron level [22,37] and then adapted to the Classification Images method [38,39]. The main concept is to place constraints on the parameter values during the estimation process. This method has been shown to be efficient for preventing the overfitting problem inherent in maximum likelihood estimation when processing a large number of parameters. In the present case, overfitting would generate a noisy ACI, which would thus describe mainly random noise, not the underlying mechanism involved in the classification. The direct consequence would be that this model would closely fit the data on which it is trained but would not be able to predict responses to novel stimuli.

The introduction of a smoothness prior allows us to reduce noise in the classification image method by applying low-pass smoothing in a principled manner and therefore to minimize overfitting [40,41]. We characterize the smoothness of the ACI with the quadratic form $Q\left(\underset{\_}{\theta }\right)=Q\left(\underset{\_}{\beta }\right)={\underset{\_}{\beta }}^{T}\underset{\_}{\underset{\_}{L}}\underset{\_}{\beta }$, where $\underset{\_}{\underset{\_}{L}}$ is the Laplacian matrix, encoding the adjacency between the pixels of the spectrotemporal representation [37,42]. This function assumes higher values when neighboring weights in the ACI markedly differ. The smoothness is assumed to be equal over the two dimensions of the ACI (time and frequency). Note that this assumption can be more or less plausible depending on the sampling rates of the time and frequency axes. We can address the issue by using two separate smoothing priors. However, doing so would dramatically increase the computational cost in our case.

Rather than maximizing the log-likelihood, we maximize the log-posterior, thereby yielding a maximum a posteriori (MAP) estimate:

$${\hat{\underset{\_}{\theta }}}_{MAP}=\underset{\underset{\_}{\theta }}{\text{argmax}}\left[L\left(\underset{\_}{\theta }\right)+\lambda \cdot Q\left(\underset{\_}{\theta }\right)\right]$$ (3)

**Fig 2:**
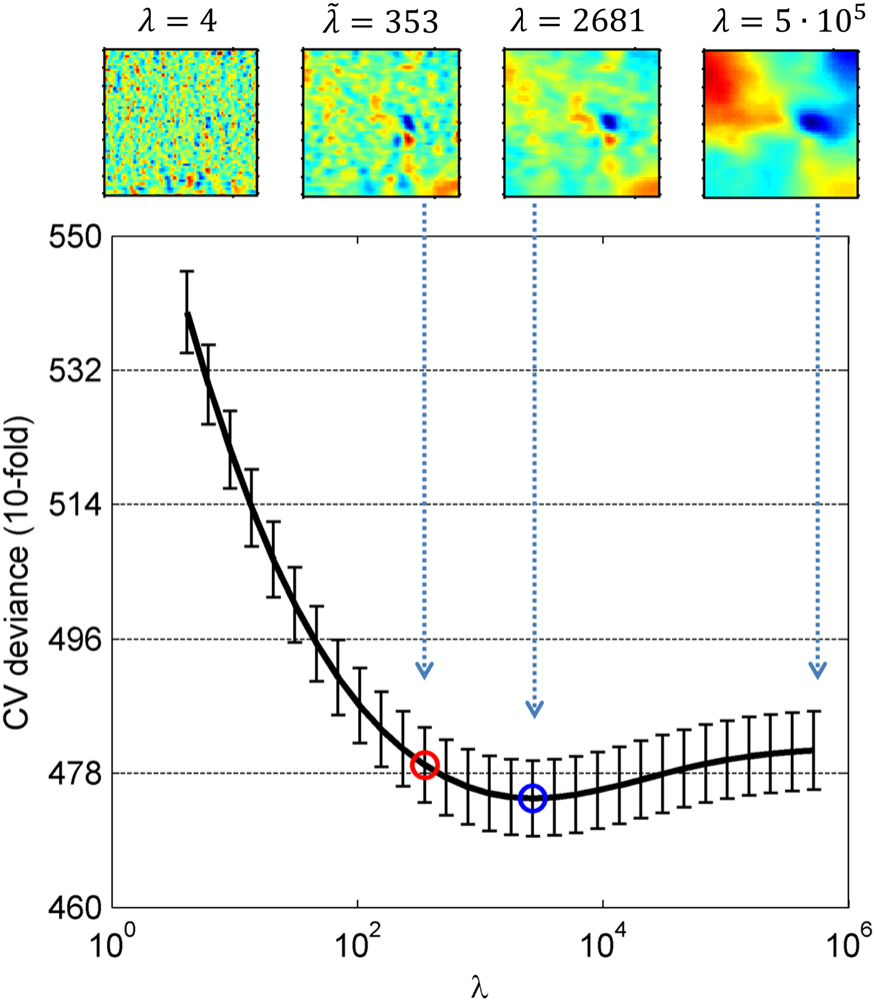
CV deviance of the penalized GLM for participant #17 as a function of regularization parameter *λ*. The minimum value of the CVD function is indicated with a blue circle, and the optimal lambda $\tilde{\lambda }$ is indicated with a red circle. Examples of ACIs obtained with different values for this participant are shown below.

In this context, $\lambda \cdot Q\left(\underset{\_}{\theta }\right)$ is called the “Regularizer” and corresponds to our a priori beliefs regarding the model parameters. In this equation, it acts as a penalty term, assigning a high cost for strong deviations between neighboring parameters and thus enforcing smoother estimates. *λ* is called the “hyperparameter” as it does not appear in the model Equation (2) but affects the final estimate. It controls the tradeoff between fitting the data well and obtaining a smooth ACI by determining the degree to which roughness should be penalized (higher penalty for increasing values of *λ*; for *λ* = 0, we recover the non-penalized maximum likelihood estimate). Given $\underset{\_}{c}$, $\underset{\_}{\underset{\_}{X}}$, and $\underset{\_}{r}$, it is possible to estimate the model parameters ${\hat{\underset{\_}{\theta }}}_{MAP}$ associated with a given hyperparameter value *λ* using the function *glmfitqp* developed by Mineault in his MATLAB toolbox *fitglmqp*. Examples of such *λ*-dependent ACIs are shown in Fig. 2.

### Lambda optimization

However, consistent with the literature on STRF and CI estimations using penalized likelihood [37,38,40], we do not want to presume an a priori value for *λ*. Instead, we aim to determine how much smoothing is appropriate based on our data. Because we aim for the ACIs to be generalizable to an independent dataset, models corresponding to different smoothing values are evaluated with a cross-validation approach, and we determine an optimal regularization parameter, denoted $\tilde{\lambda }$, according to this criterion.

#### Individual optimization

For example, in our previous paper [26], we computed a 10-fold cross-validation for a wide range of *λ* values by randomly partitioning the dataset each time between a “training” set and “test” set, thereby estimating the model parameters on the training set (9,000 trials) through MAP for all considered *λ* values, as explained previously, and then comparing the predicted responses on the test set (1,000 trials) to the actual responses of the participant. The same procedure was followed in the present study, except that the match between predicted and observed responses was assessed by computing the deviance of each fitted model applied to the test set. This deviance is a more natural measure of goodness-of-fit in the case of GLMs [41]. In this manner, each *λ* value is associated with a corresponding cross-validated deviance *CVD(λ)*, which is the mean deviance for the 10 cross-validations (see Fig. 2 for an example of one participant). For small values of *λ*, the estimate is overfitted and unreliable for predicting unseen data, thus generating a high CVD. As *λ* increases, the true estimate emerges (with a subsequent decrease in CVD) and is finally smoothed out for high *λ* values. This final step generally corresponds to a slow increase in CVD.

Thus, an optimal hyperparameter could be found by selecting the model that yields a minimum CVD value, that is to say, the degree of smoothness of the ACI that allows the most accurate predictions of unseen data. However, the increase of this function for high lambda values is sometimes relatively small, thus causing an oversmoothing of the estimate. Thus, we rather selected the smallest *λ* value at which the CVD becomes smaller than the minimum plus one standard deviation, denoted $\tilde{\lambda }$. A similar criterion, the “one-standard-error” rule, is presented in [43] and is implemented with the MATLAB function *lassoglm*.

#### Conjoint optimization

Gathering data from N participants enables the selection of the lambda values conjointly. Rather than estimating N distinct optimal hyperparameters, we can select a single value $\tilde{\lambda }$ to apply to all models. Considering the sum of the individual CVDs enables the derivation of two optimal lambda values for the group identically as performed above (with the standard deviation estimated over the group of participants’ CVDs). The resulting models may not predict the answers of the participants as well as the individual models; however, a major advantage of this method is that it provides increased stability of the hyperparameter selection, even with a limited number of trials. Furthermore, a common degree of smoothing may be required to gather different images in a group analysis. The convergence and stability of the lambda optimization will be investigated below.

### Statistical tests

Because the ACI estimate is intrinsically noisy, certain quantitative measures are required to distinguish random or non-significant effects in the images from actual functional cues. In previous works, we performed a bootstrap test to identify significant observations at the individual ACI level. In practice, experimenters will typically be more interested in formulating generalizable conclusions about a population of subjects rather than a simple sum of remarks on the behavior of several single subjects. Group analyses provide a method of testing hypotheses on the probability distribution of weights from which the individual ACIs are drawn. Hence, they will allow us to make generalizations about a population from a sample. Statistical tests were conducted at the group level for two purposes: 1) to identify the significant differences between ACIs calculated under two conditions and 2) to assess the significance of the weights for one condition. Statistical tests were performed on the z-scored individual ACIs. In both cases, the tests involved as many comparisons as there are parameters in one ACI (4,374 in our case); therefore, it is important to correct for multiple comparisons [44].

When comparing ACIs between two conditions, we used a cluster-based non-parametric test. This statistical procedure, originally developed to analyze neuroimaging data [45–47], allows the correlation inherent to the natural images to be taken into account (i.e., each pixel depending on the values of the adjacent pixels). Statistical analyses were conducted using FieldTrip, an open-source MATLAB toolbox developed for processing electrophysiological data [48]. The test is performed at two statistical levels. First, a running paired t-test is performed on all participants and compares weights at each time-frequency bin between the two conditions of interest. Second, the result is corrected for multiple comparisons by thresholding the output of the (two-tailed) t-test at p<0.01 and clustering adjacent significant samples in the time-frequency space. The statistic used to describe these clusters is T_sum_, the sum of all t-values inside the cluster. A permutation-test is performed by randomly re-assigning the ACI of each individual between the two conditions (5,000 iterations in our case) to obtain an estimate of the distribution of T_sum_ under the null hypothesis. It is then possible to compare the experimental value of T_sum_ with this calculated distribution to decide whether to reject the null hypothesis given a specified alpha value.

Because this procedure applies only to comparisons between conditions, the significance of weights in one ACI was corrected using a simple false discovery rate (FDR) correction. As a cluster-based non-parametric test, this statistical technique has been widely used for addressing the problem of multiple comparisons in neuroimaging studies [49]. More precisely, in this case, a t-value was calculated for each pixel corresponding to the hypothesis that the corresponding weight is significantly different from zero across participants. This result was then corrected by keeping the probability of type I error below a threshold of FDR<.01.

## Results

### Behavioral results

**Fig 3:**
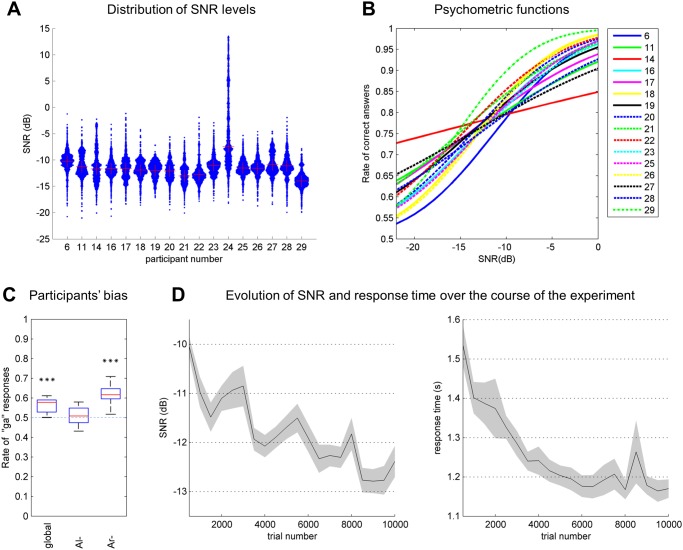
Overview of performance across all participants. A. Distribution of SNR levels for all participants (N = 17), with the mean SNR indicated with crosses. The width of each histogram is proportional to the number of trials ran at the corresponding SNR. B. Psychometric functions for participants included in the analysis (N = 16). C. Participants’ bias towards response ‘ga’ (N = 16) over the entirety of the experiment (“global”) and in conditions Al- and Ar-. Conditions with a significant bias (i.e., rate of ‘ga’ responses differing significantly from the level of 0.5 based on a random distribution, blue line) are indicated by asterisks. D. Evolution of the SNR and response time over the course of the experiment for sessions of 500 trials. Mean for the participants collectively (N = 16) with s.e.m.

Due to the extreme length of the experiment, particular care was taken to ensure comfortable listening at all times for the participants. They reported no perceived effect of excessive mental fatigue over the course of the experiment, although some participants admitted experiencing occasional and brief attention loss. As expected, participants obtained a mean correct response rate of 78.8%, as determined by the adaptive SNR algorithm. Moreover, it was possible to determine their individual performances by observing the SNR levels (given in Fig. 3A.): except for one low performer, the SNR distributions of all participants were approximately -12 dB (mean = -11.8 ± 0.9 dB), although the individual variations were quite large, with standard deviations ranging from 1.32 to 2.47 dB. Participant 24 did not achieve a stable 79% point and was therefore excluded from analysis, as noted earlier. To characterize the participants’ performances more precisely, we estimated their individual psychometric functions (the rate of correct responses as a function of the SNR) by fitting a cumulative normal distribution of unknown threshold and slope [50,51]. The results are shown in Fig. 3B. The similarity between psychometric functions suggests that all participants included in the analysis formed a homogeneous group of listeners, at least in terms of the SNR level at which they correctly categorize 79% of all stimuli.

A slight but significant bias of all participants toward response ‘ga’ can be observed from the mean rate of ‘ga’ responses (Fig. 3C, p<10^-4^). An analysis of variance (ANOVA) was conducted, with proportions of ‘ga’ responses as the dependent variable and context (Al- or Ar-) and the SNR level (high or low SNR) as the within-subject factors, thus revealing a significant main effect of context (F(1,15) = 78.65, p<10^-10^): the proportions of ‘ga’ responses were higher in context Ar- (61.2%) than in context Al- (50.3%). Similarly, we obtained a below-significance trend of SNR level (F(1,15) = 2.97, p = 0.09), with a low SNR generating a lower bias (54.7%) compared with a high SNR (56.8%). There was no significant interaction effect between these two factors (p = 0.62). The bias toward response ‘ga’ is linked to the participants’ scores, with a higher percentage of correct answers linked to stimulus ‘Arga’ (89.4%) compared with stimulus ‘Arda’ (67.2%), whereas the percentages are extremely similar between stimuli ‘Alda’ (79.1%) and ‘Alga’ (79.4%).

Additionally, the characteristics and distributions of responses are not time-stationary but evolved over the course of the experiment. Thus, a clear progressive facilitation effect was observed in terms of both the reaction time (decreasing from 1.53 s in the first session to 1.17 s in the final session, p<10^-5^) and SNR level (from -10.0 to -12.4 dB, p<10^-5^) (see Fig. 3D). Thus, at the end of the experiment, each listener was performing the task more rapidly and more efficiently. Similarly, the mean bias tends to disappear over the course of the experiment, from 60.2% of ‘ga’ responses in the first session to 50.6% in the final session (p<10^-6^). However, this effect can be considered a direct consequence of the decreasing SNR, as low SNR levels have been shown to be associated with lower biases.

### Obtained auditory classification images

Seven ACIs were derived for each of the 16 participants: in addition to the “global” statistical model that considered all responses from one participant (10,000 trials), we estimated the model parameters from different subsets of the data (each of 5,000 trials) to attempt to disentangle the effects of several factors on the ACI. Six conditions were defined according to the context (target beginning with Al- or Ar-), the trial number (the first 5,000 trials or the last 5,000 trials), and the SNR (the 5,000 highest SNRs or the 5,000 lowest SNRs). In the “global” condition, one individual hyperparameter $\tilde{\lambda }$ was selected to fit the model parameters ($\underset{\_}{b}$ and the ACI $\underset{\_}{\beta }$). For proper averaging of the ACIs of multiple participants, we also selected a conjoint hyperparameter ($\tilde{\lambda }$ = 1,191), as explained in the Materials and Methods section. These values and goodness of fit (the minimum of the CVD curve) are systematically reported in the corresponding figures. To enable comparison between participants and conditions, in each ACI the weights are divided by their maximum absolute value.

**Fig 4:**
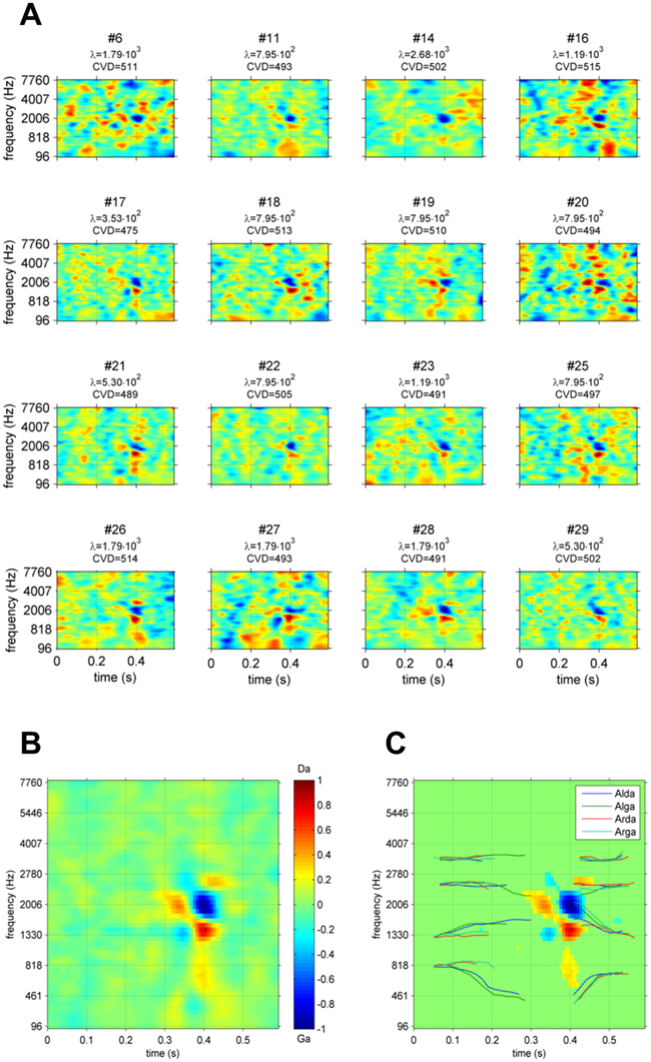
Global ACIs. A. Individual ACI estimated on 10,000 trials for all 16 participants. $\tilde{\lambda }$ and CVD are indicated below each image. B. Mean ACI for the participants collectively (estimated with λ = 1,191). C. Same ACI, with non-significant weights set to 0 (corrected t-test, FDR<0.01) and formant trajectories superimposed. In each ACI, weights are divided by their maximum absolute value.

The “global” ACIs for each participant are shown in Fig. 4A. As expected, there were slight differences in smoothness due to the variation of the regularization parameter,$\tilde{\lambda }$. The difference in terms of contrast is also notable, with some ACIs exhibiting a large number of maxima (as for participant #6), whereas others appear to be more focused (e.g., participant #19). Nevertheless, all participants exhibit a similar pattern of weights in a small region of times ranging from 300 to 470 ms and frequencies ranging from 1,300 to 2,800 Hz. This pattern becomes clearer for the mean ACI over all participants (Fig. 4B.). A statistical analysis revealed that the seven most distinct acoustic cues were all composed of positive or negative weights significantly different from zero (corrected t-test, FDR = 0.01). The significant weights are shown in Fig. 4C.

**Fig 5:**
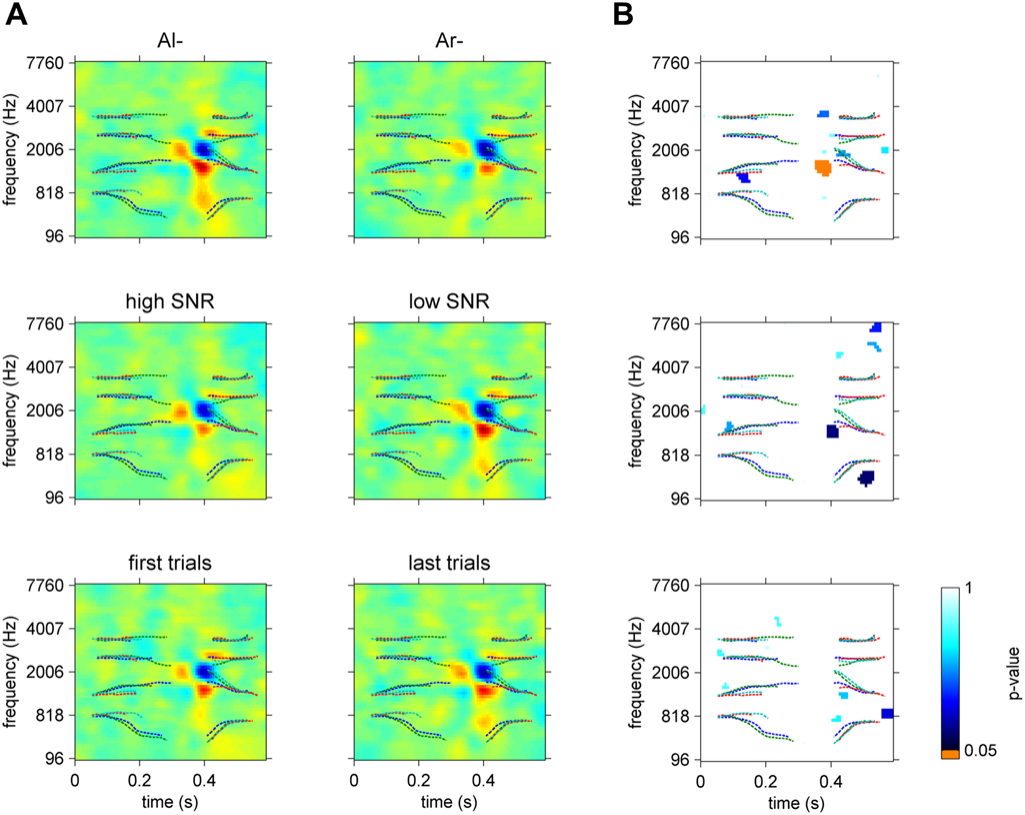
ACIs estimated on subsets of the data. A. Mean ACI for the 16 participants collectively, for conditions Al-, Ar-, high SNR, low SNR, firsttrials and lasttrials. Each individual ACI was estimated on 5,000 trials, with $\tilde{\lambda }$ = 1.191. In each ACI, the weights are divided by their maximum absolute value, and the formant trajectories are superimposed (same legend as in Fig. 6). B. Position of significant (orange) and non-significant clusters for each comparison (cluster-based non parametric test, p<0.05).

To further explore this result, we dissociated the effects of the context by estimating the model parameters separately on the 5,000 responses to targets beginning with /al/ (‘Alda’ or ‘Alga’) and on the 5,000 responses to targets beginning with /aʁ/ (‘Arda’ or ‘Arga’), with the same value of $\tilde{\lambda }$ = 1,191 as before. Differences between the two resulting ACIs are considered to reflect the non-linearities of the auditory system [52]. The “signal-specific” ACIs are shown in Fig. 5A. Notably, the distribution of weights differs slightly between both conditions.

A cluster-based nonparametric test was performed on the difference between the two ACIs to confirm this result (see Fig. 5B a paired t-test with cluster based correction, p<0.05). Indeed, one cluster appears to be significant, corresponding to a difference in the weighting of the main positive cue (p = 0.045, Tsum = 139.9).

Conversely, a similar comparison between the first 5,000 trials (condition “firsttrials”) and the last 5,000 trials (condition “lasttrials”) elicited no significant difference (Fig. 5, p>0.3, |Tsum| = 50.2). No differences were found between the 5,000 trials with the highest SNR (condition “highSNR”) and the 5,000 trials with the lowest SNR (condition “lowSNR”) (Fig. 5, p>0.15, |Tsum|<77.7).

## Discussion

In the present experiment, we used a psychoacoustic imaging method to isolate acoustic cues from the natural stimuli in a speech-in-noise categorization task. Participants were asked to perform 10,000 classifications in the presence of Gaussian noise. During each trial, they answered whether they heard /da/ or /ga/, independently of the preceding context. The accuracy rate of 78.8% for 16 participants for a SNR range of approximately -11.8 dB and their similar psychometric functions confirmed that they all successfully performed the task. Moreover, all participants included in the study demonstrated a performance improvement over time in terms of both the SNR and response time. Finally, when dissociating the participants’ answers with respect to the presented stimulus, the rate of “ga” responses was higher in context “Ar” than in context “Al”. This result may seem contradictory to that of Mann [7]. Using a continuum of synthetic “da” and “ga” varying only in the height of F3 onset, preceded by a synthetic context “Al” or “Ar”, she demonstrated that participants were more likely to answer “ga” in context “Al” and “da” in context “Ar.” This effect was interpreted as direct evidence of “compensation for coarticulation” and was reproduced in several studies. However, in the present experiment using natural stimuli, our particular utterance of “Arga” may simply be produced more distinctly and may therefore be more robust to noise than “Arda”, as suggested by the lower percentage of correct answers for the latter. This difference would account for a lower rate of “ga” responses in context “Al”. Nevertheless, this slight response bias was not an issue, as a sufficient number of responses were obtained for both types for the ACI estimation.

The calculation of ACI at the group level exhibited well-defined clusters of weights on the onsets of the F2 and F3 transitions. As has been suggested in previous studies [7,53,54], the main acoustic cues involved in this categorization task are the onsets of these two formants. Here, when there is a large amount of noise in the central negative cluster (approximately 0.4s and 2,000 Hz) corresponding to the junction between the two formants in the syllable ‘ga,’ the F2 and F3 onsets are perceived as closer than they actually are, and the target is more likely to be categorized as ‘ga.’ Conversely, when the noise is mainly distributed above or below this cluster, the target is more likely to be categorized as ‘da.’ This result also confirmed that participants were categorizing stimuli as speech sounds, not by relying on non-phonetic cues, such as prosody or intonation. As the auditory system detects variations in acoustic energy rather than absolute values, all 3 “main” acoustic cues are preceded by an inverse, smaller cue lasting approximately 0.35s, thus demonstrating an effect of temporal masking: perception of stimulus energy in a cochlear band is relative to energy at the previous time instant.

One objective of this study was to examine the specificity of an ACI to the particular utterances used in the categorization: do the positions and weightings of the acoustic cues depend on the production of speech used as targets? This question was not answered in the previous experiment involving only one recording each of “aba” and “ada.” In the present experiment, we used two productions of each target phoneme instead. To ensure that these two utterances of the same phoneme were acoustically different in a predictable manner, they were produced and presented in a situation of coarticulation, i.e., preceded by two different contexts /al/ and /aʁ/. Indeed, the production of a stop consonant is influenced by the position of the preceding context. As evidenced by the cochleograms of the 4 stimuli (Fig. 1), the two couples of allophones, although sharing a similar pattern, exhibit slight differences in the relative power and precise position of their formants (e.g., relative onset times between the two ‘da,’ F3 onset frequency between the two ‘ga’). Additionally, the perception of a phoneme can be biased by the preceding context [7,55,56]. One question that arose was the following: are those differences reflected in the ACIs? When splitting the ACIs according to the first syllable, we could reveal significant differences between the ACI in context Al- and in context Ar-. These differences are typically interpreted as nonlinearities in the auditory/speech perception system, with the processing applied to the input signal depending on the signal [52]. More specifically, the significant cluster corresponds to a difference in the weighting of the main cues: in context Ar-, participants relied less heavily on the main positive cue. The cause of this dissimilarity could not be determined with certainty because our comparison involves differences in both the targets and their contexts. A possible explanation may be that this imbalance between the positive cue and adjacent negative cue in context Ar- could correspond to a mechanism of compensation for coarticulation, as both Ar- contexts have F2 and F3 at the frequencies corresponding to those of the positive cues. Thus, the participants could perceptually mask the positive cues at the same frequency, compared with the central negative cue, by a simple spectral contrast effect [9,57].

Two other conditions were tested in addition to the Al- /Ar- contexts. The absence of significant differences between the ACIs calculated on the first and last 5,000 trials (conditions “firsttrials” and “lasttrials”) suggests that participants’ performance improvement over the course of the experiment did not rely on a modification of the listening strategy. A possible alternative mechanism would be a diminution of internal noise: listeners are more likely to provide the same answer when presented with the same stimulus twice at the end of the experiment than at the beginning. Unfortunately, the estimation of internal noise requires a two-pass experiment [58,59], which was impossible to implement here given the large number of trials.

**Fig 6:**
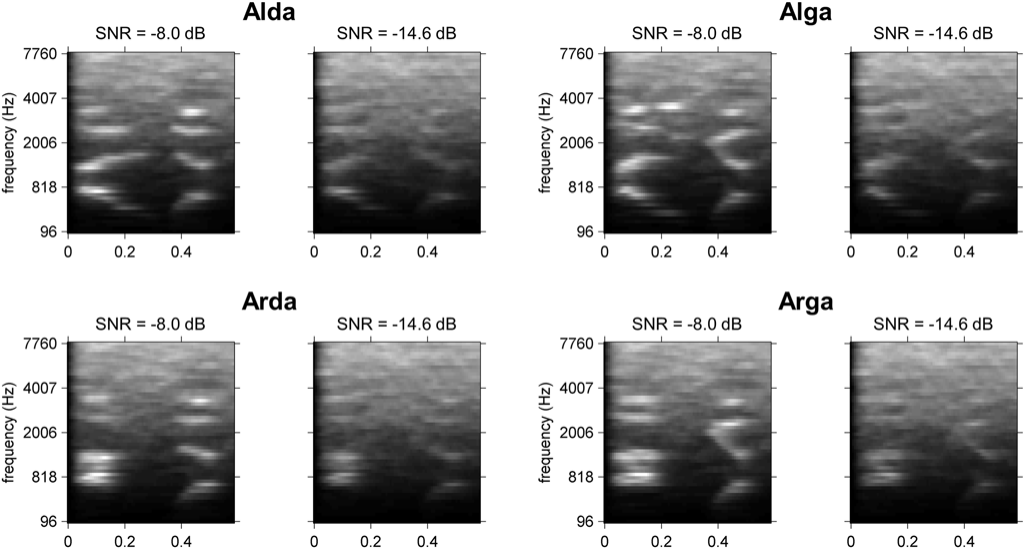
Cochleograms of the 4 signals in noise, with SNR = -8.0 dB or -14.6 dB. The parameters used for the cochleograms are described in paragraph Materials and Methods.

Finally, the non-significant comparison between conditions highSNR and lowSNR suggests that the listening strategy did not depend crucially on the level of noise during the experiment. Rather, it may rely on the same acoustic cues, regardless of whether the background noise was important. Across a series of studies, Allen and colleagues carefully studied the confusion patterns in a phoneme-recognition task as a function of SNR while linking discontinuities in the probabilities of a given answer and the robustness of the critical acoustic cues [17–19]. It may appear surprising that similar noise-dependent cues were not observed in our study. However, in our case, the range of SNR values was considerably smaller: overall, 90% of the trials were between -8.0 and -14.6 dB, whereas in the experiment conducted by Allen and colleagues, the SNR value varied from 12 to -22 dB. One may assume that no critical acoustic cues are masked in our lowSNR condition compared with the highSNR condition, as confirmed by the representations of the four signals in noise at -8.0 and -14.6 dB (Fig. 6).

In the next sections, we discuss the assumptions underlying the ACI method and possible improvements.

### Cochleogram representation

The use of a GLM does not require the noise samples in ${\underset{\_}{X}}_{i}$ to be normally distributed, thus allowing us to select from most auditory models to represent the sounds. In their 2013 article, Varnet and colleagues chose to derive their ACIs from the spectrogram of the noise. However, the spectrogram is not the most suitable representation for studying speech perception because it does not consider the specificities of processing in the outer and middle ear, such as the spacing and bandwidth of the auditory filters. Thus, we decided to use a more biologically inspired representation of speech, the cochleogram, thereby yielding a “higher-level” representation of the functional acoustic cues. Because of the quasi-logarithmic frequency axis, mimicking the resolution of the auditory system, the acoustic cues in different frequency bands should be similar in size. This similarity is important for applying the smoothness prior, which acts here as a low-pass filter. Indeed, spatial smoothing would make it impossible to detect cues of large and small sizes simultaneously, as will be discussed further below.

Nonetheless, other representations could be used. A promising approach would be to combine the ACI technique with the multiresolution model developed by Chi and colleagues [25]. This combination would yield a 3-dimensional matrix of weights reflecting the importance of different regions of the time-frequency space for the phonemic categorization.

### Are smoothness priors well adapted?

The introduction of a smoothness prior in the GLM estimation provides a means of selecting the amount of filtering to be applied to the ACI by minimizing the error predicting new data rather than applying an arbitrary degree of smoothing. This powerful tool is highly useful when estimating a matrix of parameters with unknown smoothness from a series of noisy measurements. However, the determination of the optimal spatial smoothing in a principled manner is not immune to other filtering problems, such as those described in [60]. One limitation is that if patterns of multiple scales are present, then the filtering can make the detection of both patterns simultaneously nearly impossible. In other words, our smoothness optimization introduces one assumption in the estimation process: that all relevant acoustic cues must be of similar sizes. However, the bandwidth of the auditory filters varies with their center frequencies. The cochleogram representation considers, at least partially, this differential sensitivity along the basilar membrane. Nevertheless, acoustic cues covering several auditory filters may have different sizes.

**Fig 7:**
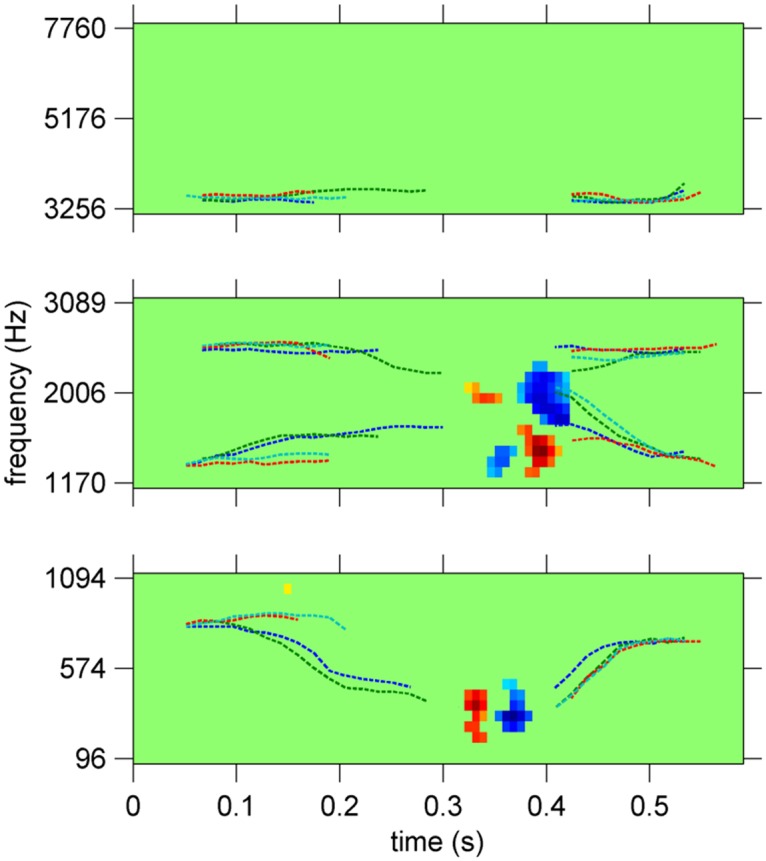
Band-limited ACIs, in low-frequency (90–1,100 Hz, $\tilde{\lambda }$ = 36, middle frequency (1,100–3,100 Hz, $\tilde{\lambda }$ = 144) and high frequency (3,100–8,000 Hz, $\tilde{\lambda }$= 144) bands. In each band, weights are divided by their maximum absolute value and formant trajectories are superimposed (same legend as in Fig. 6). Non-significant weights are set to 0 (corrected t-test, FDR<0.01).

Indeed, when dividing our frequency axis into three bands with equal numbers of parameters (low frequencies: 90–1,100 Hz; middle frequencies: 1,100–3,100 Hz; high frequencies: 3,100–8,000 Hz) and estimating three separate ACIs for each participant, we obtained different degrees of smoothing for the three frequency bands. The same acoustic cues were obtained in the middle frequency band, and no significant weight was found in the high-frequency band. Unexpectedly, a clear acoustic cue appeared in the low-frequency band, with a much lower degree of smoothing different than in the middle frequency band (Fig. 7). This small-sized low-frequency cue was not predicted by the previous studies on this task, as they focused solely on the F2-F3 transition. Thus, our band-limited ACI indicates that this simple categorization task involves the processing of several spectral and temporal cues. One possible interpretation may be found in [13]. This synthetic speech study suggests that the identity of the consonant may be affected by the synchronicity between the F1 onset and the locus of the transition. Thus, a temporal translation of the first formant might change the consonant percept, thereby explaining the presence of a temporal cue on the onset of the first formant in our ACI. This low-frequency cue was not detected during the “global” ACI estimation because the middle-frequency cues, which are of different size, more accurately predict the participants’ responses. Therefore, the CVD curve attains its minimum for the lambda value corresponding to the smoothness of the main cues, a value that is too high to render a good resolution of the secondary cues.

The presence of multiple resolutions clearly shows a limitation of the smoothness prior: cues of multiple sizes cannot be found simultaneously in a single estimate. One solution in our case would be to implement the constraint on not the smoothness but the number of cues to be detected. This adjustment could be enabled by the “sparse prior on a smooth basis” described in the work by Mineault et al. [39]. Using the same GLM, this penalization would seek to improve the accuracy of the prediction of the participants’ answers by placing a restricted number of Gaussian-shaped patterns of weights of various scales on the ACI. Moreover, in their visual experiment, Mineault and colleagues demonstrated that the sparse prior offers a more accurate prediction than the smoothness prior for a given number of trials in terms of CV deviance.

### Number of trials required

One crucial question for the application of the ACI method relates to the length of the experiment. We examined separately how the number of trials influences the hyperparameter selection and the quality of the estimated templates.

#### Number of trials required for lambda optimization

**Fig 8:**
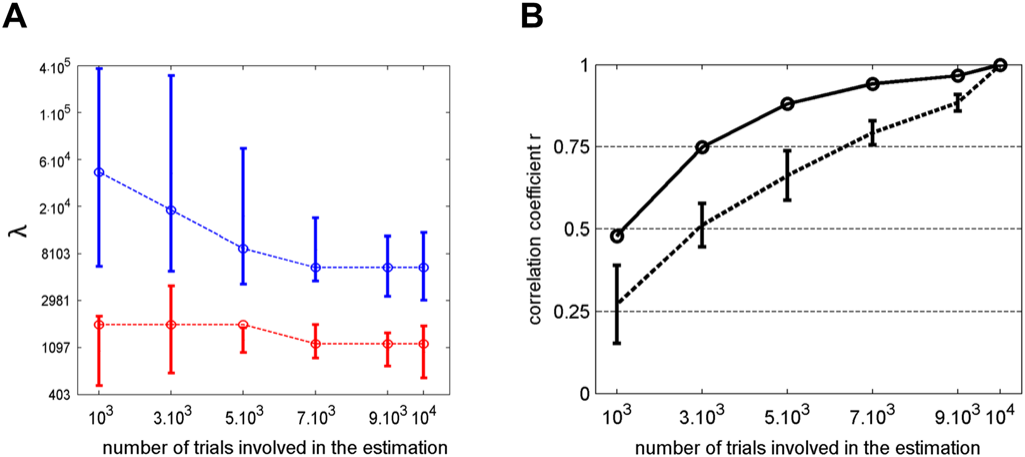
Effect of the number of trials involved in the estimation. A. Evolution of $\tilde{\lambda }$ for a different number of trials. Red circles indicate the result of the conjoint optimization, and red error bars indicate the standard deviation of the individual lambdas. Blue circles and blue error bars indicate the position of the minimum value of the CVD function for groups and individuals, respectively. B. Correlation between ACIs estimated on n trials, and final ACI (*λ* = 1,191). Dotted line: mean and standard deviation of the correlation for individual ACIs; thick line: correlation for the mean ACI for the participants collectively.

Fig. 8A. depicts the optimal lambda values obtained with different numbers of trials (in red). By definition, $\tilde{\lambda }$, the point at which the CVD becomes smaller than the minimum plus one standard deviation, is smaller than the lambda value corresponding to the minimum of the CVD curve (in blue). Both points appear to be biased toward higher values when the number of trials is insufficient to provide a reliable ACI (approximately with less than 5,000 trials). Indeed, in this case, the CVD curve does not attain a minimum but plateaus after an abrupt decrease. The participants’ response bias toward ‘ga,’ a bias that has been shown to be stronger in the first sessions of this experiment, may also affect the overestimation of $\tilde{\lambda }$. Nevertheless, the lambda selection appears to be relatively robust, even with as few as 1,000 trials. Comparatively, a selection based on the minimum of the CVD curve would perform less well in terms of both the bias and variability across participants.

#### Number of trials required for reliable ACI estimation

As a second step, we investigated the number of trials necessary to ensure a reliable estimation of the underlying template. The accuracy of one ACI was evaluated by examining its correlation with the final ACI calculated on 10,000 trials. All ACIs were estimated with *λ* = 1,191 ($\tilde{\lambda }$ for 10,000 trials). The results of the individual and mean ACIs are presented in Fig. 8B. Whereas the accuracy of individual estimates decreases steadily with a decreasing number of trials involved in the estimation, the mean ACI for all 16 participants in total remains high (r>0.75) until approximately 3,000 trials (the estimation noise being reduced by the averaging).

Overall, the data collected from 16 participants enable the number of trials required from each participant to be reduced to approximately 3,000 by selecting the lambda value conjointly and considering the mean image for all participants. More importantly, this multi-participants study offers the opportunity to apply statistical tests at the group level rather than at the individual level.

#### Future directions

Here we have described a new methodology to investigate the way in which the human speech perception system achieves fast and efficient categorization of phonemes in noise. An appealing application would be to combine the ACI approach with electrophysiological measurements, such as EEG recordings or intracranial recordings. This would offer a direct way to identify the neural correlates of acoustic cue detection during speech perception. Furthermore the similarities with statistical methods employed in time-frequency analyses of electrophysiological data [22,48] would make it possible to draw parallel analyses of neural and behavioral responses. For the time being however, the duration of the experiment would constitute a major impediment.

Two plausible solutions to overcome this problem should be considered in future studies. At present we can obtain a good level of precision for individual images using 5000 trials, as mentioned above. A solution to further reduce this number of trials would be to introduce some additional a priori knowledge about the acoustic cues to be sought. For example, if we assume that the cues could be well represented by a limited number of Gaussian bumps we can use a GLM with sparse priors on a smooth basis, which is far more powerful, as done by Mineault et al. [39]. Alternatively, future studies investigating the neural signatures of speech categorization using the ACI approach could explore the recording of Speech Auditory Brainstem Response (ABR) [61,62]. This type of experiment typically requires a few thousand presentations of speech stimuli. In this context, one could derive the ACI directly from the ABR instead of the behavioral response of the participant.

## Conclusion

We demonstrated how the GLM with smoothness prior approach, combined with a cluster-based test, provides a reliable approach for investigating the acoustic cues involved in a specific phoneme categorization task. Through the example of a da/ga categorization in the contexts of Ar- and Al-, we confirmed that listeners relied on the F2 and F3 onsets. We also demonstrated that the perceived timing of F1influences the categorization. Finally, the method was proven precise enough to track fine modifications in the weighting of the different cues depending on the specific utterance presented. Three constraints of the ACI technique and possible solutions were discussed: the dependency on the sound representation, the choice of the prior, and the number of trials required. Despite these limitations, such a psychoacoustic method, which involves no prior knowledge of the spectrotemporal locations of the acoustic cues being sought, offers a valuable insight into the mechanisms of speech perception. Additionally, the ACI technique can be combined with statistical tests at a group level, thus making it a powerful tool to investigate hypotheses on human speech recognition.

## Supporting Information

S1 Table Results of preliminary screening tests for all participants. (XLSX)
